# Search for patterns by combining cosmic-ray energy and arrival directions at the Pierre Auger Observatory

**DOI:** 10.1140/epjc/s10052-015-3471-0

**Published:** 2015-06-20

**Authors:** A. Aab, P. Abreu, M. Aglietta, E. J. Ahn, I. Al Samarai, I. F. M. Albuquerque, I. Allekotte, J. Allen, P. Allison, A. Almela, J. Alvarez Castillo, J. Alvarez-Muñiz, R. Alves Batista, M. Ambrosio, A. Aminaei, L. Anchordoqui, S. Andringa, C. Aramo, V. M. Aranda, F. Arqueros, H. Asorey, P. Assis, J. Aublin, M. Ave, M. Avenier, G. Avila, N. Awal, A. M. Badescu, K. B. Barber, J. Bäuml, C. Baus, J. J. Beatty, K. H. Becker, J. A. Bellido, C. Berat, M. E. Bertaina, X. Bertou, P. L. Biermann, P. Billoir, S. Blaess, M. Blanco, C. Bleve, H. Blümer, M. Boháčová, D. Boncioli, C. Bonifazi, R. Bonino, N. Borodai, J. Brack, I. Brancus, A. Bridgeman, P. Brogueira, W. C. Brown, P. Buchholz, A. Bueno, S. Buitink, M. Buscemi, K. S. Caballero-Mora, B. Caccianiga, L. Caccianiga, M. Candusso, L. Caramete, R. Caruso, A. Castellina, G. Cataldi, L. Cazon, R. Cester, A. G. Chavez, A. Chiavassa, J. A. Chinellato, J. Chudoba, M. Cilmo, R. W. Clay, G. Cocciolo, R. Colalillo, A. Coleman, L. Collica, M. R. Coluccia, R. Conceição, F. Contreras, M. J. Cooper, A. Cordier, S. Coutu, C. E. Covault, J. Cronin, A. Curutiu, R. Dallier, B. Daniel, S. Dasso, K. Daumiller, B. R. Dawson, R. M. de Almeida, M. De Domenico, S. J. de Jong, J. R. T. de Mello Neto, I. De Mitri, J. de Oliveira, V. de Souza, L. del Peral, O. Deligny, H. Dembinski, N. Dhital, C. Di Giulio, A. Di Matteo, J. C. Diaz, M. L. Díaz Castro, F. Diogo, C. Dobrigkeit, W. Docters, J. C. D’Olivo, A. Dorofeev, Q. Dorosti Hasankiadeh, M. T. Dova, J. Ebr, R. Engel, M. Erdmann, M. Erfani, C. O. Escobar, J. Espadanal, A. Etchegoyen, P. Facal San Luis, H. Falcke, K. Fang, G. Farrar, A. C. Fauth, N. Fazzini, A. P. Ferguson, M. Fernandes, B. Fick, J. M. Figueira, A. Filevich, A. Filipčič, B. D. Fox, O. Fratu, U. Fröhlich, B. Fuchs, T. Fujii, R. Gaior, B. García, S. T. Garcia Roca, D. Garcia-Gamez, D. Garcia-Pinto, G. Garilli, A. Gascon Bravo, F. Gate, H. Gemmeke, P. L. Ghia, U. Giaccari, M. Giammarchi, M. Giller, C. Glaser, H. Glass, M. Gómez Berisso, P. F. Gómez Vitale, P. Gonçalves, J. G. Gonzalez, N. González, B. Gookin, J. Gordon, A. Gorgi, P. Gorham, P. Gouffon, S. Grebe, N. Griffith, A. F. Grillo, T. D. Grubb, F. Guarino, G. P. Guedes, M. R. Hampel, P. Hansen, D. Harari, T. A. Harrison, S. Hartmann, J. L. Harton, A. Haungs, T. Hebbeker, D. Heck, P. Heimann, A. E. Herve, G. C. Hill, C. Hojvat, N. Hollon, E. Holt, P. Homola, J. R. Hörandel, P. Horvath, M. Hrabovský, D. Huber, T. Huege, A. Insolia, P. G. Isar, I. Jandt, S. Jansen, C. Jarne, M. Josebachuili, A. Kääpä, O. Kambeitz, K. H. Kampert, P. Kasper, I. Katkov, B. Kégl, B. Keilhauer, A. Keivani, E. Kemp, R. M. Kieckhafer, H. O. Klages, M. Kleifges, J. Kleinfeller, R. Krause, N. Krohm, O. Krömer, D. Kruppke-Hansen, D. Kuempel, N. Kunka, D. LaHurd, L. Latronico, R. Lauer, M. Lauscher, P. Lautridou, S. Le Coz, M. S. A. B. Leão, D. Lebrun, P. Lebrun, M. A. Leigui de Oliveira, A. Letessier-Selvon, I. Lhenry-Yvon, K. Link, R. López, A. Lopez Agüera, K. Louedec, J. Lozano Bahilo, L. Lu, A. Lucero, M. Ludwig, M. Malacari, S. Maldera, M. Mallamaci, J. Maller, D. Mandat, P. Mantsch, A. G. Mariazzi, V. Marin, I. C. Mariş, G. Marsella, D. Martello, L. Martin, H. Martinez, O. Martínez Bravo, D. Martraire, J. J. Masías Meza, H. J. Mathes, S. Mathys, J. Matthews, J. A. J. Matthews, G. Matthiae, D. Maurel, D. Maurizio, E. Mayotte, P. O. Mazur, C. Medina, G. Medina-Tanco, R. Meissner, M. Melissas, D. Melo, A. Menshikov, S. Messina, R. Meyhandan, S. Mićanović, M. I. Micheletti, L. Middendorf, I. A. Minaya, L. Miramonti, B. Mitrica, L. Molina-Bueno, S. Mollerach, M. Monasor, D. Monnier Ragaigne, F. Montanet, C. Morello, M. Mostafá, C. A. Moura, M. A. Muller, G. Müller, S. Müller, M. Münchmeyer, R. Mussa, G. Navarra, S. Navas, P. Necesal, L. Nellen, A. Nelles, J. Neuser, P. Nguyen, M. Niechciol, L. Niemietz, T. Niggemann, D. Nitz, D. Nosek, V. Novotny, L. Nožka, L. Ochilo, A. Olinto, M. Oliveira, N. Pacheco, D. Pakk Selmi-Dei, M. Palatka, J. Pallotta, N. Palmieri, P. Papenbreer, G. Parente, A. Parra, T. Paul, M. Pech, J. Pȩkala, R. Pelayo, I. M. Pepe, L. Perrone, E. Petermann, C. Peters, S. Petrera, Y. Petrov, J. Phuntsok, R. Piegaia, T. Pierog, P. Pieroni, M. Pimenta, V. Pirronello, M. Platino, M. Plum, A. Porcelli, C. Porowski, R. R. Prado, P. Privitera, M. Prouza, V. Purrello, E. J. Quel, S. Querchfeld, S. Quinn, J. Rautenberg, O. Ravel, D. Ravignani, B. Revenu, J. Ridky, S. Riggi, M. Risse, P. Ristori, V. Rizi, W. Rodrigues de Carvalho, I. Rodriguez Cabo, G. Rodriguez Fernandez, J. Rodriguez Rojo, M. D. Rodríguez-Frías, D. Rogozin, G. Ros, J. Rosado, T. Rossler, M. Roth, E. Roulet, A. C. Rovero, S. J. Saffi, A. Saftoiu, F. Salamida, H. Salazar, A. Saleh, F. Salesa Greus, G. Salina, F. Sánchez, P. Sanchez-Lucas, C. E. Santo, E. Santos, E. M. Santos, F. Sarazin, B. Sarkar, R. Sarmento, R. Sato, N. Scharf, V. Scherini, H. Schieler, P. Schiffer, D. Schmidt, F. G. Schröder, O. Scholten, H. Schoorlemmer, P. Schovánek, A. Schulz, J. Schulz, J. Schumacher, S. J. Sciutto, A. Segreto, M. Settimo, A. Shadkam, R. C. Shellard, I. Sidelnik, G. Sigl, O. Sima, A. Śmiał kowski, R. Šmída, G. R. Snow, P. Sommers, J. Sorokin, R. Squartini, Y. N. Srivastava, S. Stanič, J. Stapleton, J. Stasielak, M. Stephan, A. Stutz, F. Suarez, T. Suomijärvi, A. D. Supanitsky, M. S. Sutherland, J. Swain, Z. Szadkowski, M. Szuba, O. A. Taborda, A. Tapia, M. Tartare, A. Tepe, V. M. Theodoro, C. Timmermans, C. J. Todero Peixoto, G. Toma, L. Tomankova, B. Tomé, A. Tonachini, G. Torralba Elipe, D. Torres Machado, P. Travnicek, E. Trovato, M. Tueros, R. Ulrich, M. Unger, M. Urban, J. F. Valdés Galicia, I. Valiño, L. Valore, G. van Aar, P. van Bodegom, A. M. van den Berg, S. van Velzen, A. van Vliet, E. Varela, B. Vargas Cárdenas, G. Varner, J. R. Vázquez, R. A. Vázquez, D. Veberič, V. Verzi, J. Vicha, M. Videla, L. Villaseñor, B. Vlcek, S. Vorobiov, H. Wahlberg, O. Wainberg, D. Walz, A. A. Watson, M. Weber, K. Weidenhaupt, A. Weindl, F. Werner, A. Widom, L. Wiencke, B. Wilczyńska, H. Wilczyński, M. Will, C. Williams, T. Winchen, D. Wittkowski, B. Wundheiler, S. Wykes, T. Yamamoto, T. Yapici, G. Yuan, A. Yushkov, B. Zamorano, E. Zas, D. Zavrtanik, M. Zavrtanik, I. Zaw, A. Zepeda, J. Zhou, Y. Zhu, M. Zimbres Silva, M. Ziolkowski, F. Zuccarello

**Affiliations:** Centro Atómico Bariloche and Instituto Balseiro (CNEA-UNCuyo-CONICET), San Carlos de Bariloche, Argentina; Centro de Investigaciones en Láseres y Aplicaciones, CITEDEF and CONICET, Villa Martelli, Buenos Aires, Argentina; Departamento de Física, FCEyN Universidad de Buenos Aires y CONICET, Buenos Aires, Argentina; IFLP, Universidad Nacional de La Plata and CONICET, La Plata, Argentina; Instituto de Astronomía y Física del Espacio (CONICET-UBA), Buenos Aires, Argentina; Instituto de Física de Rosario (IFIR), CONICET/U.N.R. and Facultad de Ciencias Bioquímicas y Farmacéuticas U.N.R., Rosario, Argentina; Instituto de Tecnologías en Detección y Astropartículas (CNEA, CONICET, UNSAM) and National Technological University, Faculty Mendoza (CONICET/CNEA), Mendoza, Argentina; Instituto de Tecnologías en Detección y Astropartículas (CNEA, CONICET, UNSAM), Buenos Aires, Argentina; Observatorio Pierre Auger, Malargüe, Argentina; Observatorio Pierre Auger and Comisión Nacional de Energía Atómica, Malargüe, Argentina; Universidad Tecnológica Nacional - Facultad Regional Buenos Aires, Buenos Aires, Argentina; University of Adelaide, Adelaide, SA Australia; Centro Brasileiro de Pesquisas Fisicas, Rio de Janeiro, RJ Brazil; Faculdade Independente do Nordeste, Vitória da Conquista, Brazil; Universidade de São Paulo, Escola de Engenharia de Lorena, Lorena, SP Brazil; Instituto de Física de São Carlos, Universidade de São Paulo, São Carlos, SP Brazil; Instituto de Física, Universidade de São Paulo, São Paulo, SP Brazil; Universidade Estadual de Campinas, IFGW, Campinas, SP Brazil; Universidade Estadual de Feira de Santana, Feira de Santana, Brazil; Universidade Federal da Bahia, Salvador, BA Brazil; Universidade Federal de Pelotas, Pelotas, RS Brazil; Universidade Federal do ABC, Santo André, SP Brazil; Instituto de Física, Universidade Federal do Rio de Janeiro, Rio de Janeiro, RJ Brazil; Universidade Federal Fluminense, EEIMVR, Volta Redonda, RJ Brazil; Rudjer Bošković Institute, 10000 Zagreb, Croatia; Faculty of Mathematics and Physics, Institute of Particle and Nuclear Physics, Charles University, Prague, Czech Republic; Institute of Physics of the Academy of Sciences of the Czech Republic, Prague, Czech Republic; Palacky University, RCPTM, Olomouc, Czech Republic; Institut de Physique Nucléaire d’Orsay (IPNO), Université Paris 11, CNRS-IN2P3, Orsay, France; Laboratoire de l’Accélérateur Linéaire (LAL), Université Paris 11, CNRS-IN2P3, Orsay, France; Laboratoire de Physique Nucléaire et de Hautes Energies (LPNHE), Universités Paris 6 et Paris 7, CNRS-IN2P3, Paris, France; Laboratoire de Physique Subatomique et de Cosmologie (LPSC), Université Grenoble-Alpes, CNRS/IN2P3, Grenoble, France; Station de Radioastronomie de Nançay, Observatoire de Paris, CNRS/INSU, Nançay, France; SUBATECH, École des Mines de Nantes, CNRS-IN2P3, Université de Nantes, Nantes, France; Bergische Universität Wuppertal, Wuppertal, Germany; Karlsruhe Institute of Technology - Campus South - Institut für Experimentelle, Kernphysik (IEKP), Karlsruhe, Germany; Karlsruhe Institute of Technology - Campus North - Institut für Kernphysik, Karlsruhe, Germany; Karlsruhe Institute of Technology - Campus North - Institut für Prozessdatenverarbeitung und Elektronik, Karlsruhe, Germany; Max-Planck-Institut für Radioastronomie, Bonn, Germany; RWTH Aachen University, III. Physikalisches Institut A, Aachen, Germany; Universität Hamburg, Hamburg, Germany; Universität Siegen, Siegen, Germany; Università di Milano and Sezione INFN, Milan, Italy; Università di Napoli “Federico II” and Sezione INFN, Napoli, Italy; Università di Roma II “Tor Vergata” and Sezione INFN, Roma, Italy; Università di Catania and Sezione INFN, Catania, Italy; Università di Torino and Sezione INFN, Torino, Italy; Dipartimento di Matematica e Fisica “E. De Giorgi” dell’Università del Salento and Sezione INFN, Lecce, Italy; Dipartimento di Scienze Fisiche e Chimiche dell’Università dell’Aquila and INFN, L’Aquila, Italy; Gran Sasso Science Institute (INFN), L’Aquila, Italy; Istituto di Astrofisica Spaziale e Fisica Cosmica di Palermo (INAF), Palermo, Italy; INFN, Laboratori Nazionali del Gran Sasso, Assergi, L’Aquila Italy; Osservatorio Astrofisico di Torino (INAF), Università di Torino and Sezione INFN, Torino, Italy; Benemérita Universidad Autónoma de Puebla, Puebla, Mexico; Centro de Investigación y de Estudios Avanzados del IPN (CINVESTAV), Mexico, Mexico; Universidad Michoacana de San Nicolas de Hidalgo, Morelia, Michoacan Mexico; Universidad Nacional Autonoma de Mexico, Mexico, D.F., Mexico; IMAPP, Radboud University Nijmegen, Nijmegen, Netherlands; KVI - Center for Advanced Radiation Technology, University of Groningen, Groningen, Netherlands; Nikhef, Science Park, Amsterdam, Netherlands; ASTRON, Dwingeloo, Netherlands; Institute of Nuclear Physics PAN, Krakow, Poland; University of Łódź, Łódź, Poland; Laboratório de Instrumentação e Física Experimental de Partículas - LIP and Instituto Superior Técnico - IST, Universidade de Lisboa - UL, Lisbon, Portugal; ’Horia Hulubei’ National Institute for Physics and Nuclear Engineering, Bucharest-Magurele, Romania; Institute of Space Sciences, Bucharest-Magurele, Romania; Physics Department, University of Bucharest, Bucharest, Romania; University Politehnica of Bucharest, Bucharest, Romania; Experimental Particle Physics Department, J. Stefan Institute, Ljubljana, Slovenia; Laboratory for Astroparticle Physics, University of Nova Gorica, Nova Gorica, Slovenia; Universidad Complutense de Madrid, Madrid, Spain; Universidad de Alcalá, Alcalá de Henares, Madrid Spain; Universidad de Granada and C.A.F.P.E., Granada, Spain; Universidad de Santiago de Compostela, Santiago de Compostela, Spain; School of Physics and Astronomy, University of Leeds, Leeds, UK; Case Western Reserve University, Cleveland, OH USA; Colorado School of Mines, Golden, CO USA; Colorado State University, Fort Collins, CO USA; Colorado State University, Pueblo, CO USA; Department of Physics and Astronomy, City University of New York, New York, USA; Fermilab, Batavia, IL USA; Louisiana State University, Baton Rouge, LA USA; Michigan Technological University, Houghton, MI USA; New York University, New York, NY USA; Northeastern University, Boston, MA USA; Ohio State University, Columbus, OH USA; Pennsylvania State University, University Park, PA USA; Enrico Fermi Institute, University of Chicago, Chicago, IL USA; University of Hawaii, Honolulu, HI USA; University of Nebraska, Lincoln, NE USA; University of New Mexico, Albuquerque, NM USA

## Abstract

Energy-dependent patterns in the arrival directions of cosmic rays are searched for using data of the Pierre Auger Observatory. We investigate local regions around the highest-energy cosmic rays with $E \ge 6 \times 10^{19}$ eV by analyzing cosmic rays with energies above $E \ge 5 \times 10^{18}$ eV arriving within an angular separation of approximately 15$^{\circ }$. We characterize the energy distributions inside these regions by two independent methods, one searching for angular dependence of energy-energy correlations and one searching for collimation of energy along the local system of principal axes of the energy distribution. No significant patterns are found with this analysis. The comparison of these measurements with astrophysical scenarios can therefore be used to obtain constraints on related model parameters such as strength of cosmic-ray deflection and density of point sources.

## Introduction

The long-standing question about the origin and nature of the ultra-high energy cosmic rays (UHECRs) is yet unanswered. Presumably, UHECRs are charged nuclei of extragalactic origin. They are deflected in extragalactic magnetic fields and the magnetic field of the Milky Way such that their arrival directions may not point back to their sources [1]. The structure, strength, and origin of these cosmic magnetic fields are open questions in astrophysics as well [2, 3]. Consequently, UHECRs can also be considered to be probes of the magnetic fields they traverse [4, 5] as the deflections lead to energy-dependent patterns in their arrival directions, and an analysis of such patterns may allow for conclusions on the strength and structure of the fields.

The Pierre Auger Observatory [6, 7] is currently the largest experiment dedicated to observations of UHECRs. In 2007, we reported evidence for a correlation of events with energies above 60 EeV ($1 \,\hbox {EeV} = 10^{18}$ eV) with the distribution of nearby extragalactic matter [8, 9]. An update of the analysis yielded a correlation strength which is reduced compared to the initial result [10]. Further searches for anisotropy using variants of autocorrelation functions [11] yielded no statistically-significant deviation from isotropic scenarios. Following this observation, constraints on the density of point sources and magnetic fields have been reported [12]. Also a direct search for magnetically-induced alignment in the arrival directions of cosmic rays assuming they were protons has been performed without uncovering so-called multiplet structures beyond isotropic expectations [13].

Nevertheless, if the highest-energy cosmic rays with $E>60$ EeV are tracers of their sources and even if their deflection in magnetic fields is dependent on their nuclear charges, some of the lower-energy cosmic rays in a region around them may be of the same origin. From deflections both in extragalactic magnetic fields and the magnetic field of the Milky Way, their distribution of arrival directions may show energy-dependent patterns. In particular a circular ‘blurring’ of the sources is expected from deflection in turbulent magnetic fields, while energy dependent linear structures are expected from deflection in coherent magnetic fields.

In this report, we investigate the local regions around cosmic rays with $E \ge 60$ EeV by analyzing cosmic rays with energies above $E = 5$ EeV arriving within an angular separation of 0.25 rad. The lower energy cut just above the ankle is motivated by the assumption that the selected cosmic rays are predominantly of extragalactic origin. The angular separation cut has been optimized from simulation studies and will be explained below.

We use two methods to characterize the energy distributions inside the local regions. In one method we study energy-energy correlations between pairs of cosmic rays depending on their angular separation from the center of the region. With this measurement we search for signal patterns expected from particle deflection in turbulent magnetic fields. In the second method we decompose the directional energy distribution of the cosmic rays along its principal axes. This general decomposition method imposes no requirement on the sign of the cosmic-ray charge, or the charge itself. Beyond measuring the strength of collimation along principal axes, the axis directions of the individual regions around the highest-energy cosmic rays potentially reveal local deflection patterns due to magnetic fields.

Both methods were originally studied in particle physics, and were referred to as energy-energy correlations and thrust observables, respectively [14, 15]. Simulations of their application in cosmic-ray physics have demonstrated the capability to reveal effects from coherent and turbulent magnetic fields [16, 17].

This paper is structured as follows. The observables of the energy-energy correlations and the principal-axis analysis are defined in Sect. 2. Their response to structure potentially expected from deflection in magnetic fields is illustrated using a simplified model in Sect. 3. The measured distributions of the observables using data of the surface detector of the Pierre Auger Observatory are presented in Sect. 4. In Sect. 5, we first analyze the directional characteristics of the measured principal axes by studying their reproducibility. We then present a comparison of the measurements with an astrophysical model of UHECR origin and propagation, and determine constraints on the source density, and the strength of cosmic-ray deflection as the two dominant model parameters.

## Definitions

In this section we introduce the main components used for the measurement. We first define the local regions in which we analyze the cosmic-ray energies and arrival directions. We then explain the energy-energy correlation observable and its angular dependence. Finally, we present the method of calculating the principal axes of the energy distribution which results in the three values to characterize the strength of collimation along each axis, and the directions of the axes themselves.

### Region of interest

The observables used here are calculated from the events detected in a bounded region in the sky, here denoted as ‘region of interest’ (ROI). To minimize the statistical penalty from multiple tries, we do not scan the entire sky but investigate a limited number of ROIs located around events with an energy above 60 EeV. This energy cut is motivated by the limitation of the propagation distance by, e.g., the GZK effect [18, 19] and corresponds to the energy used in the AGN correlation analysis [8]. The size of the ROIs, i.e. the maximum angular separation of a UHECR belonging to the ROI to the center of the ROI, is set to 0.25 rad. To choose these values we simulated the UHECR propagation in magnetic fields with the UHECR simulation tool PARSEC [20] for different strengths of the deflection and source density. The simulations were analyzed with varying choices of parameters. The chosen values maximize the power of the observables to discriminate between scenarios with strong deflections and isotropic scenarios [21, 22]. To avoid a possible bias of the characterization of the ROI, we exclude the cosmic ray seeding the ROI from the calculation of the observables.

### Energy-energy correlations

Energy-energy correlations (EECs) are used to obtain information on the turbulent part of galactic and extragalactic magnetic fields [16]. The concept of the EEC was originally developed for tests of quantum chromodynamics (QCD) [14]. The Energy-energy correlation $\varOmega _{ij}$ is calculated for every pair of UHECRs *i*, *j* within a ROI using

1 $$\begin{aligned} \varOmega _{ij}= \frac{(E_i-\langle E(\alpha _i) \rangle )\,(E_j-\langle E(\alpha _j) \rangle ) }{E_i \, E_j}. \end{aligned}$$

Here $E_i$ is the energy of the UHECR *i* with the angular separation $\alpha _i$ to the center of the ROI. $\langle E_i(\alpha _i) \rangle$ is the average energy of all UHECRs at the angular separation $\alpha _i$ from the center of the ROI.

The cosmic rays in a ROI can be separated into a signal fraction, whose arrival direction is correlated with the energy, and an isotropic background fraction. The values of $\varOmega _{ij}$ can be positive or negative depending on the cosmic-ray pair having energies above or below the average energies. An angular ordering is measured in the following sense. A pair of cosmic rays, one being above and the other below the corresponding average energy, results in a negative correlation $\varOmega _{ij} < 0$. This is a typical case for a background contribution. A pair with both cosmic rays having energies above or below the average energy at their corresponding angular separation gives a positive correlation $\varOmega _{ij} > 0$. Here both signal and background pairs are expected to contribute. As the correlations are determined as a function of the opening angle to the center of the ROI, circular patterns can be found that are expected from turbulent magnetic deflections which are sometimes viewed as random-walk propagation.

We present the angular distribution of the EEC as the average distribution of all ROIs. Each value $\varOmega _{ij}$ is taken into account twice, once at the angular separation $\alpha _i$ and once at $\alpha _j$.

### Principal axes

To further characterize energy-dependent patterns within each individual ROI, we calculate the three principal axes of the energy distribution which we denote as $\mathbf {n}_{k=1,2,3}$. For this we successively maximize the quantity

2 $$\begin{aligned} T_k = \max _{\mathbf {n}_k} \left( \frac{\sum _i |\omega _i^{-1}\; \mathbf {p}_{i}\cdot \mathbf {n}_k|}{\sum _i |\omega _i^{-1}\; \mathbf {p}_i|} \right) \end{aligned}$$

with respect to the axes $\mathbf {n}_{k}$ starting with $k=1$. Here $\mathbf {p}_i$ is the cosmic-ray momentum and $\omega _i$ the corresponding exposure of the detector [23] in the direction of particle *i*. The values of $T_{k=1,2,3}$ quantify the strength of the collimation of the particle momenta along each of the three axes $\mathbf {n}_{k=1,2,3}$ of the principal system. We denote $T_{k=1,2,3}$ as thrust observables following previous studies of perturbative QCD in particle collisions [15, 24].

For $k = 1$ the quantity $T_1$ is called the ‘thrust’ and consequently the first axis of the principal system $\mathbf {n}_1$ is called ‘thrust axis’. For the second axis the additional condition $\mathbf {n}_1 \perp \mathbf {n}_2$ is used in Eq. (2). The resulting value $T_2$ is denoted as ‘thrust major’, the axis as ‘thrust-major axis’. Finally, the third quantity $T_3$ is called ‘thrust minor’ with corresponding ‘thrust-minor axis’. For the thrust-minor axis $\mathbf {n}_3$ it is $\mathbf {n}_1 \perp \mathbf {n}_2 \perp \mathbf {n}_3$ which renders the maximization in Eq. (2) trivial. From this definition follows $T_1 > T_2 > T_3$.

In arbitrarily defined spherical coordinates $(r, \phi , \theta )$ with orthonormal basis $(\mathbf {e}_r, \mathbf {e}_\phi , \mathbf {e}_\theta )$ and the observer at the center, the momenta of the particles at the high energies considered here can be written as $\mathbf {p}_i = \vert E_i \vert \mathbf {e}_{r_i}$ with the energy $E_i$ and the radial unit vector $\mathbf {e}_{r_i}$ in the arrival direction of particle *i*. The thrust axis is thus the radial unit vector $\mathbf {e}_r$ pointing to the local barycenter of the energy distribution, and the thrust value is a measure for the energy-weighted strength of clustering of the events. For no dispersion of the particles in the region it takes on the value $T_1 = 1$, whereas for an isotropic distribution in a circular region the expectation value of $T_1$ depends dominantly on the size of the ROI [22].

The thrust-major and thrust-minor axes can consequently be written as

3 $$\begin{aligned} \mathbf {n}_{2}= & {} \cos {\xi _{2}} \, \mathbf {e}_\phi + \sin {\xi _{2}} \, \mathbf {e}_\theta \end{aligned}$$

4 $$\begin{aligned} \mathbf {n}_{3}= & {} \cos {\xi _{3}} \, \mathbf {e}_\phi + \sin {\xi _{3}} \, \mathbf {e}_\theta \end{aligned}$$

with the angles $\xi _2$ and $\xi _3 = 90^\circ + \xi _2$ between the corresponding axes and the vector $\mathbf {e}_\phi$. Using this together with Eq. (2), the thrust-major $T_2$ becomes maximal if $\mathbf {n}_2$ is aligned with a linear distribution of UHECR arrival directions. The thrust-major axis thus points along threadlike structures in the energy distribution of UHECRs. As the thrust minor axis is chosen perpendicular to $\mathbf {n}_1$ and $\mathbf {n}_2$ it has no physical meaning beyond its connection to the thrust-major axis. However, the thrust-minor $T_3$ gives meaningful information as it denotes the collimation strength perpendicular to the thrust-major axis.

Note that in a perfect isotropic scenario, the energy distribution within the plane defined by $\mathbf {n}_2$ and $\mathbf {n}_3$ exhibits perfect symmetry. The values of $T_2$ and $T_3$ are approximately equal, and the axis directions are accidental. However, even with a small signal contribution beyond an isotropic background, the circular symmetry in the $(\mathbf {n}_2 , \mathbf {n}_3)$ plane is broken giving rise to unequal values of $T_2$ and $T_3$. In addition, the direction of the thrust-major axis then reveals valuable directional information. This directional information can be compared to the direction of deflection obtained in a multiplet analysis [13]. However, in contrast to the multiplet analysis the principal axes analysis does not require a uniform charge of the cosmic rays. Its sensitivity is driven by the total deflection amount.

## Benchmark distributions for coherent and turbulent magnetic fields

For obtaining a general understanding of the energy-energy correlations and the thrust observables, we use simple scenarios of cosmic-ray deflections in magnetic fields to demonstrate resulting distributions. First we describe the procedure for simulating mock data representing cosmic-ray deflection in turbulent and coherent magnetic fields. For different quantitative mixtures of these field types we then present the distributions of the energy-energy correlations and finally discuss the resulting thrust distributions.

### Simulation procedure

To demonstrate the sensitivity of the observables to deflections expected from magnetic fields, we simulate a ROI with UHECRs in a simplified scenario. The deflection in cosmic magnetic fields is supposed to result in two different kinds of patterns in the arrival direction of the UHECRs. First, if the UHECR’s trajectory resembles a directed random walk, a symmetric blurring of the source is expected. Second, if the particles are deflected in large-scale coherent fields, e.g. in the Milky Way, an energy ordering of the UHECRs in threadlike multiplets is expected.

**Fig. 1:**
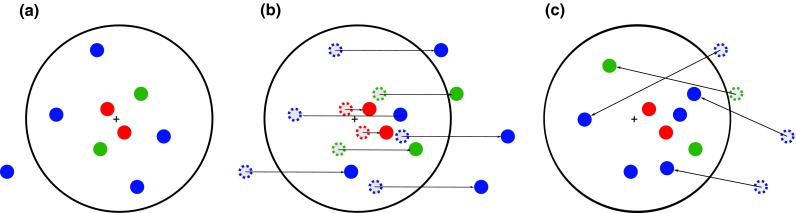
Generation of anisotropically distributed UHECRs in a region of interest. **a** First, UHECRs are distributed symmetrically around the center of the ROI using a Fisher distribution with energy dependent concentration parameter according to Eq. (6). **b** The UHECRs are then deflected in one direction using Eq. (8). **c** UHECRs deflected outside of the ROI are moved to a random position inside the region

Here we model the distribution of UHECRs in a region around the source as a superposition of both effects. Events in this region of interest are generated in three steps as sketched in Fig. 1. First, the UHECRs are distributed around the center of the ROI following a Fisher distribution [25] with probability density

5 $$\begin{aligned} f(\alpha ,\kappa ) = \frac{\kappa }{4\pi \, \sinh {\kappa }} e^{(\kappa \, \cos {\alpha )}} \end{aligned}$$

for angle $\alpha$ between cosmic ray and center of the ROI. The Fisher distribution can be considered here as the normal distribution on the sphere. The concentration parameter $\kappa$ is chosen with an energy dependence that emulates the deflection in turbulent magnetic fields as

6 $$\begin{aligned} \kappa = C_\text {T}^{-2} E^{2}. \end{aligned}$$

For small deflections the distribution resembles a Rayleigh distribution where $\kappa$ is related to the root-mean-square $\delta _{\text {RMS}}$ of the deflection angles by $\kappa = \delta _{\text {RMS}}^{-2}$ and thus

7 $$\begin{aligned} \delta _{\text {RMS}} \simeq \frac{C_\text {T}}{E}. \end{aligned}$$

A value of $C_\text {T} = 1 \,\mathrm{rad} \,\mathrm{EeV}$ is equivalent to an RMS of the deflection angle $\delta _{\text {RMS}} = 5.7^{\circ }$ for 10 EeV particles. For example, using the usual parametrization for deflections in turbulent magnetic fields [26, 27] this corresponds to the expected deflection of 10 EeV protons from a source at a distance $D \approx 16 \,\hbox {Mpc}$ propagating through a turbulent magnetic field with coherence length $\Lambda \approx 1 \,\mathrm{Mpc}$ and strength $B \approx 4nG$.

Second, a simple model for the deflection in coherent magnetic fields is added on top of the model for turbulent magnetic fields used above. Here the individual cosmic rays are deflected in one direction by an angle $\alpha$ that depends on the energy of the particles according to

8 $$\begin{aligned} \alpha = C_\text {C}\, E^{-1} \end{aligned}$$

where the parameter $C_\text {C}$ is used to model the strength of the coherent deflection. The procedure is illustrated in Fig. 1b.

Third, particles deflected outside the region of interest are added as a background to keep the number of particles in this setup constant (cf. Fig. 1c). The energies of all events are chosen following a broken power law with spectral index $\gamma _1 = -2.7$ below 40 EeV and $\gamma _2 = -4.2$ above 40 EeV to be comparable with the observed cosmic-ray energy spectrum [28].

### Response of the energy-energy correlation

**Fig. 2:**
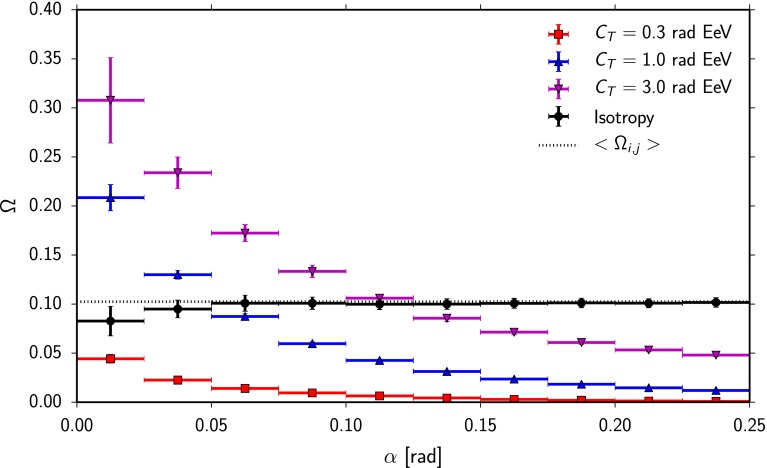
Response of the EEC to typical deflection patterns from simulations of three different turbulent deflection strengths with $C_\text {T}=0.3 \,\mathrm{rad}\,\mathrm{EeV}$ (*red squares*), $C_\text {T}=1 \,\mathrm{rad}\, \mathrm{EeV}$ (*blue upward triangles*) and $C_\text {T}=3 \,\mathrm{rad}\, \mathrm{EeV}$ (*magenta downward triangles*). The *dashed line* marks the isotropic expectation value according to Eq. (9); *black circles* denote the result from simulation of isotropically distributed UHECRs

The EEC distributions resulting from simulated scenarios using the three values for the turbulent deflection strength $C_\text {T} = 0.3, 1.0, 3.0 \,\mathrm{rad}\, \mathrm{EeV}$ are shown in Fig. 2. As the EEC is expected to provide only minor sensitivity to coherent deflections [16] $C_\text {C} = 0$ is used here. For each scenario 50 realizations of an ROI with 300 UHECRs have been used, which is approximately the number of UHECRs in a low-coverage region of the measurement presented in Sect. 5. All scenarios are compared with the result for an isotropic distribution of UHECRs. Without structure in the arrival directions of UHECRs, the EEC distribution is flat with an expectation value

9 $$\begin{aligned} \left\langle \varOmega _{ij} \right\rangle = \left\langle {\frac{{\left( {{E_i} - \left\langle E \right\rangle } \right) {} \left( {{E_j} - \left\langle E \right\rangle } \right) }}{{{E_i} {E_j}}}} \right\rangle =\left( 1- \left\langle E \right\rangle \left\langle \frac{1}{E}\right\rangle \right) ^2. \end{aligned}$$

For a source signal the typical signature is an increase towards small angles, as can be seen in Fig. 2. With increasing angular separation the UHECRs average energies decrease, and so do the differences between the UHECR energies and their corresponding average [Eq. (1)]. Consequently, the values of $\varOmega _{ij}$ can become small in contrast to a scenario where all UHECR energies contribute at every angular scale. The shape of the EEC distribution in response to a source signal depends on the deflection pattern. In general it can be seen that a small deflection causes an increase only in the innermost bins, while a larger deflection will smear this signature over the whole ROI.

### Response of the principal-axes analysis

**Fig. 3:**
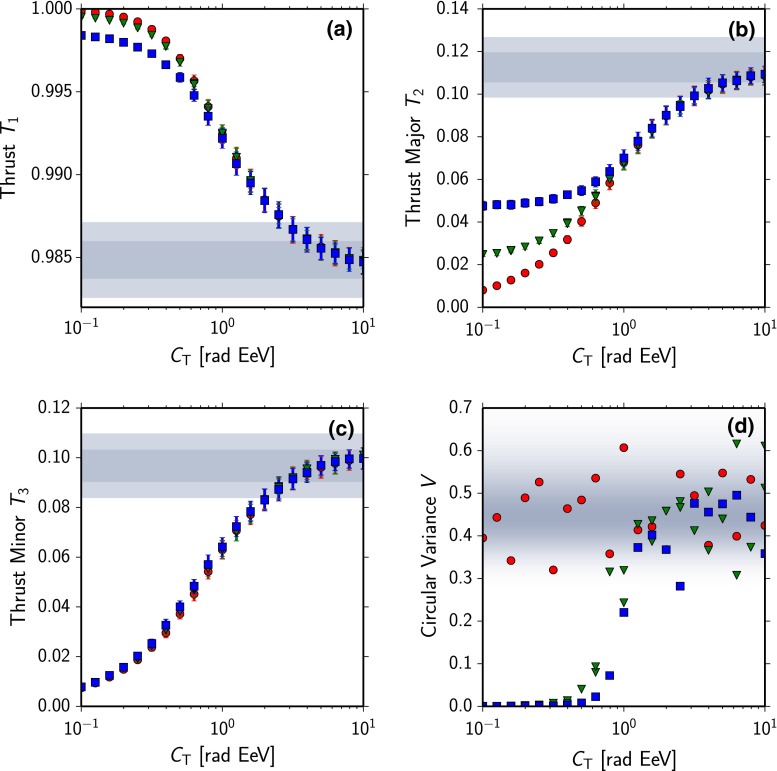
Response of the thrust observables to typical deflection patterns. **a**–**c** Mean and spread of the observables $T_{1,2,3}$ as a function of the strength of the deflection in turbulent magnetic fields $C_\text {T}$. *Red circles* correspond to no directed deflection, *green triangles* to $C_\text {C} = 0.5$ rad EeV and *blue squares* to $C_\text {C} = 1.0$ rad EeV. The *shaded area* corresponds to the $1\sigma$ and $2\sigma$ expectations of the observables for an isotropic distribution of cosmic rays. **d** Circular variance of the thrust-major axes calculated in the simulations in 100 ROIs. *Gray shading* corresponds to the probability density of the expectation value of the circular variance of uniformly-distributed directions

In Fig. 3a–c the mean and spread of the thrust observables $T_{1,2,3}$ of 100 realizations of the ROI at each point in the explored parameter space are shown. We used $C_\text {T} =$ 0.1–10 rad EeV, without coherent deflection, and alternatively with $C_\text {C}=0.5$ rad EeV as well as $C_\text {C}=1.0$ rad EeV.

All three observables are sensitive to a symmetric blurring of the source. For increasing $C_\text {T}$ the distribution of cosmic rays in the ROI becomes isotropic, and the observables approach the corresponding expectation value. The value of the thrust major and thrust minor for strong patterns is here below the expectation for no patterns, as the particles are concentrated in the center of the ROI. The thrust minor, Fig. 3c, does not depend on the strength of coherent deflection, as the width of the blurring is determined here only by the strength of $C_\text {T}$.

When measuring a thrust-major axis of an individual ROI, we also want to determine the stability of the axis direction. As explained in Sect. 2, the thrust major-axis is located in the plane tangential to a sphere around the observer, and provides a directional characteristic on the sky. We quantify the stability of the axis using the circular variance *V* derived in the specialized statistics for directional data (e.g. [29, 30]). The direction of the thrust-major axis $\mathbf {n}_{2,i}$ in a region of interest *i* is defined by the angle $\theta _i$ between the axis and the local unit vector $\mathbf {e_\phi }$ in spherical coordinates with $\theta _i \in [0 \ldots \pi )$.

To calculate the circular variance *V* from the *n* observations $\theta _i$, first the $\theta _i$ are transformed to angles on the full circle by $\theta ^*_i = \ell \cdot \theta _i$ with $\ell = 2$ owing to the symmetry of the thrust-major axis. With

10 $$\begin{aligned} C = \sum _{i=1}^n \cos \theta ^*_i,\quad S = \sum _{i=1}^n \sin \theta ^*_i \end{aligned}$$

the resultant length *R* is defined as

11 $$\begin{aligned} R = \sqrt{C^2 + S^2}. \end{aligned}$$

Based on the resultant length *R* in Eq. (11) the circular variance *V* of a sample of size *n* is defined as

12 $$\begin{aligned} V = 1 - \left( \frac{R}{n}\right) ^{1/\ell ^2}. \end{aligned}$$

In contrast to the variance in linear statistics, *V* is limited to the interval [0, 1]. The circular variance is a consistent measure for the concentration of observations on periodic intervals with $V=0$ for data from a single direction and $V=1$ for perfectly dispersed data. Even in the limit $n \ll \infty$ a value $V < 1$ is also expected for non-directed data as perfect dispersion is unlikely in a random sample.

To demonstrate the strength of correlation of the axes with the direction of deflection in the simulation we use the circular variance *V* among the simulated sample as a measure. The resulting values for the 100 simulated scenarios at every point of the aforementioned parameter space are shown in Fig. 3d. In case of zero coherent deflection, and also in case of strong blurring of the sources, no stable axis is found. For small blurring of the sources, the variance between the directions is zero, if there is coherent deflection.

## Measurement

For the measurement of the observables we selected events above 5 EeV recorded with the surface detector of the Pierre Auger Observatory up to March 19, 2013. We require that the zenith angle of the events is smaller than 60$^{\circ }$ and that the detector stations surrounding the station with the highest signal are active [7]. 30,664 events are included in the analysis; 70 fulfill the conditions $E \ge 60 \,\mathrm{EeV}$ and are at least 0.25 rad inside the field of view of the Pierre Auger Observatory and therefore seed an ROI.

In order to estimate the uncertainty on the measurement, we repeatedly vary the energy and arrival directions of all events detected with the Pierre Auger Observatory above $E = 3 \,\mathrm{EeV}$ and $\theta < 60^{\circ }$ within their experimental uncertainties and repeat the calculation of the observables with the new values. The mean and spread of the resulting distributions then serve as measured observables and their corresponding uncertainty. The energy resolution of the surface detector is 16 % [31] and the angular resolution of the SD is better than $1^{\circ }$ for energies above 5 EeV [32]. The selected ROIs are kept fixed to the original positions in all repetitions. Because of the decreasing spectrum, the number of events in the analysis increases as more events propagate above the lower energy threshold than vice versa. To keep the number of events in the uncertainty analysis fixed, the 30,664 events with the highest energy after variation are selected.

**Fig. 4:**
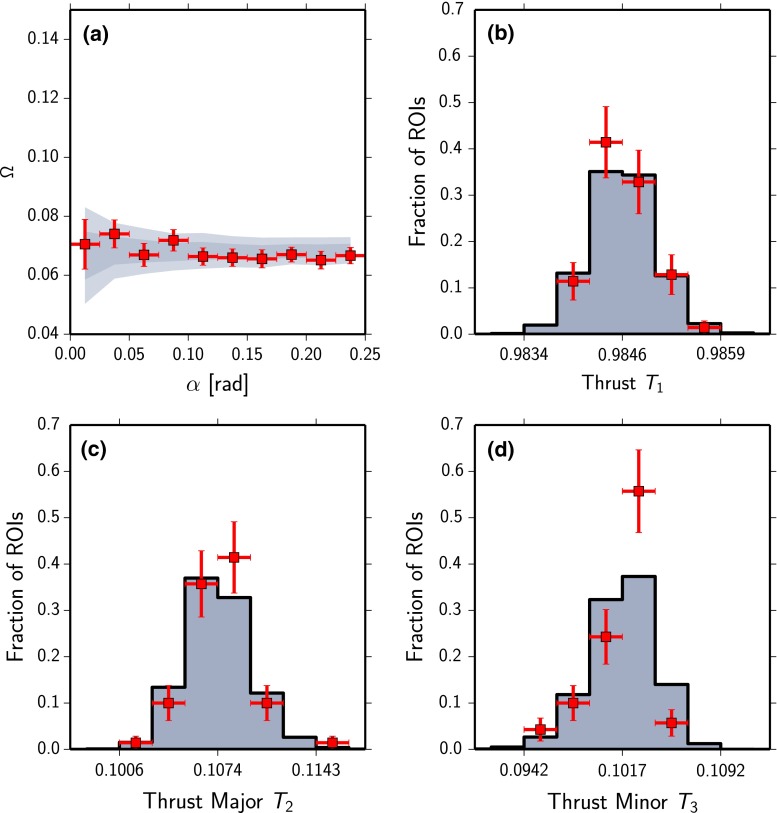
Measurement of the **a** energy-energy correlation $\varOmega$ and **b**–**d** thrust observables $T_{1,2,3}$ with the Pierre Auger Observatory (*red squares* and *error bars*). The measurements are compared to distributions without structure in the arrival directions of UHECRs (*gray distributions*)

In Fig. 4 the distributions of the measured EEC and thrust observables are shown together with the distributions expected from isotropic arrival directions of UHECRs. The goodness-of-fit of the measurements compared to expected distributions without structure in the arrival directions of UHECRs, using a $\chi ^2$ test, yields *p*-values which are all above $p=0.2$ except for the thrust minor distribution with $p(T_3)=0.01$. Note that the *p*-values for $T_3$ results from a lack of signal-like regions in the data which are expected to broaden the distribution. The measured distributions of all four observables reveal thus no local patterns in the arrival directions of UHECRs.

**Fig. 5:**
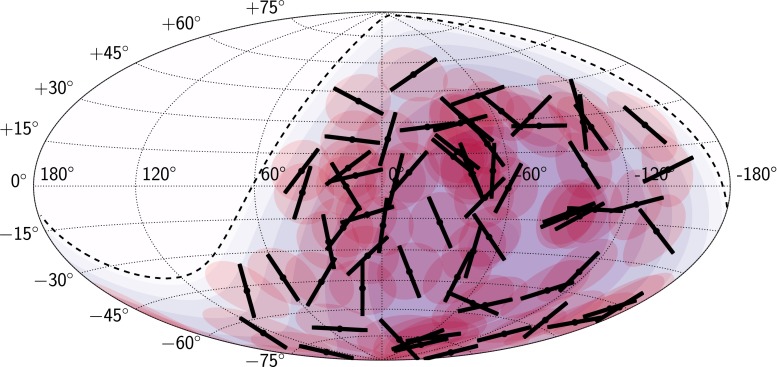
Hammer projection of the map of principal axes of the directional energy distribution in galactic coordinates. The *red shaded areas* represent the regions of interest. *Black lines* denote the second principal axes (thrust-major axes) $\mathbf {n}_2$, *black dots* mark the positions of the thrust axes $\mathbf {n}_1$. The *blue shading* indicates the exposure of the Pierre Auger Observatory; *the dashed line* marks the extent of its field of view

From the principal-axes analysis, a map of the thrust-major axes is derived which is shown in Fig. 5. If not trivial, these axes correspond to the direction of preferred cosmic-ray deflections. This question is further studied in the following section.

## Discussion

In this section we first continue with analysing the directions of the thrust axes shown as a sky map in Fig. 5. The aim is to search for any individual ROI with signal contributions, e.g. cosmic rays from a point source, by testing the reproducibility of the axis direction. We will then compare the measured distributions of the energy-energy correlations and the thrust values in Fig. 4 with astrophysical simulations obtained with the PARSEC Monte Carlo generator. Using these comparisons, limits on the strength of the deflection of the UHECRs in extragalactic magnetic fields and the density of point sources of UHECRs are derived.

### Reproducibility of the axes measurement

We further investigate the directional information shown by the thrust-major axes of the individual ROIs in Fig. 5. From the simplified simulations in Sect. 3 we saw that thrust-major directions are reproducible in repeated experiments for scenarios where coherent deflections contribute, and turbulent deflections are not too large. In additional simulation studies it was shown that evidence for anisotropy could sometimes be found in reproducibility of axis directions even when the thrust scalar values were consistent with isotropy [22]. Hence, analysis of the directions of the thrust-major axes could potentially reveal further information.

As we have obtained a single set of measured UHECR data at this point in time, we perform here a stability test on subsets of the data in the following sense. If the measured thrust-major direction obtained in a single ROI is related to a deflection pattern reasonably constant in time then the analysis of subsets of the measured data should also reflect this pattern. As only a fraction of the ROIs may contain such a deflection pattern we perform tests of reproducibility on each ROI individually.

We first define the ROIs as before using all available data. We then split the dataset into *n* independent subsamples and compare the directions $\mathbf {n}_{2,j=1} \ldots \mathbf {n}_{2,j = n}$ obtained in each subsample for every individual region of interest. A low variability of directions in the subsets of the data provides evidence for a non-triviality of the thrust-major axis and consequently for an anisotropic distribution of UHECRs.

The optimal choice for the number of subsamples to split the data into is not known a priori. On the one hand, a large number of *n* maximizes the number of repeated experiments. On the other hand, as the total number of UHECRs is fixed, $n = 2$ maximizes the number of UHECRs in every subsample. We investigated the choice of *n* using simulations of the simplified model described in Sect. 3. The test power to distinguish regions of interest containing 600 anisotropically distributed UHECRs from regions with isotropically distributed UHECRs using the circular variance *V* reaches a plateau for $n \gtrsim 12$.

The dependence of the results and their variance with random splits of the data set into 12 parts was investigated. The observed axis directions shown in Fig. 5 were not reproducible in subsets of the data with this analysis. No evidence for a non-triviality of the axes was thus found.

### Limits on propagation parameters

**Fig. 6:**
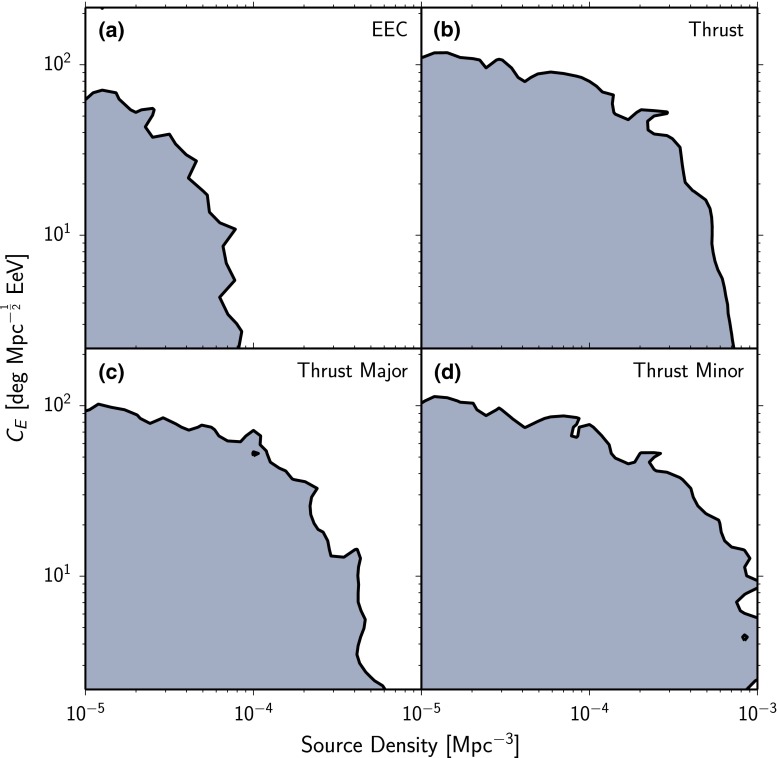
95 % $CL_S$ limits on the strength of the deflection of cosmic-ray protons $C_\text {E}$ [cf. Eq. (13) and (6) ff.] and density of point sources $\rho$ in simulations using the PARSEC software [20] from the analysis of the **a** energy-energy correlations, **b** thrust, **c** thrust-major and **d** thrust-minor distributions. The *gray areas* are excluded by the measurements

A prime value of the measurements lies in their ability to constrain UHECR propagation scenarios. We outline the procedure to derive limits on scenario parameters using a simple model for extragalactic propagation of protons based on parameterizations as implemented in version 1.2 of the PARSEC software [20]. Although this model is likely too coarse to allow definite conclusions on the sources of UHECRs, it includes at least qualitatively the effects influencing patterns in the UHECR distributions. Its fast computability allows a scan of a large range of parameter combinations in the source density and the strength of the deflection in the extragalactic magnetic field, thus limiting these important parameters within this model. The procedure to obtain limits from the measurements reported in this paper as outlined here can be applied to any other model.

The PARSEC software simulates ultra-high energy protons by calculating the probability-density function (pdf) to observe a cosmic ray for discrete directions and energies using parameterizations for energy losses and energy-dependent deflections. In the calculations, energy losses of the UHECRs from interaction with extragalactic-photon backgrounds, effects from the expansion of the universe and deflection in extragalactic magnetic fields are accounted for using parameterizations. To account for deflections in the galactic magnetic field, the calculated pdf is transformed using matrices derived from backtracked UHECRs using the CRT software [33].

As model for the galactic magnetic field, we use here the model proposed by Jansson and Farrar [34, 35]. For the random field we assume Kolmogorov turbulences with a coherence length $L_\text {c} = 60\,\mathrm{pc}$ and a maximum wavelength $L_\text {max} \simeq 260\,\mathrm{pc}$. We use only one realization of the random component of the model in all simulations. The directions in the simulations are discretized into 49,152 equal-area pixels following the HEALPix layout [36]. The energy is discretized into 100 log-linear spaced bins ranging from $10^{18.5}$ to $10^{20.5}$ eV. Both choices result in angular and energy bins smaller than the corresponding measurement errors.

We simulated scenarios with unstructured point sources with density $\rho$ and strength of the deflection of the cosmic rays

13 $$\begin{aligned} C_\text {T} = C_\text {E} \sqrt{D} \end{aligned}$$

with distance *D* of the source. We scanned the parameter range $C_\text {E} =2-200^{\circ }\mathrm{Mpc}^{-1/2} \mathrm{Eev}$ and source densities up to $\rho =1 \times 10^{-3}\,\mathrm{Mpc}^{-3}$. We considered contributions from sources up to a distance $D_{\max } = 2 \mathrm{Gpc}$. At every point of the parameter space we simulated sets of 200 pseudo experiments with the same number of events as in the measurement presented in Sect. 4.

Since the sources of the UHECRs are randomly distributed and have a maximum injection energy $E_{\max } = 1000 \,\mathrm{EeV}$, some realizations do not include sources within 43 Mpc, the maximum propagation distance of the most energetic particle in this analysis. Due to the continuous energy loss approximation the maximum distance is here a hard limit and these simulations cannot reproduce the observed energies. To restrict the reported limits to information from the observables such scenarios are not used here. Note that within such a scenario, the necessity of a close source could be used as an additional constraint. The probability of including at least one source in a pdf set can be calculated analytically (e.g. [37]) and is higher than $96 \%$ for source densities greater than $\rho =1 \times 10^{-5} \,\mathrm{Mpc}^{-3}$. Using this argument alone, source densities with $\rho < 1 \times 10^{-7} \,\mathrm{Mpc}^{-3}$ may be disfavored. However, the inclusion of this argument only marginally modifies the reported limits.

Limits on the strength of the deflection and the density of point sources in the simulation are set using the $CL_S$ method [38, 39]. Here,

14 $$\begin{aligned} Q = -2 \log \frac{\mathcal {L}_\text {a}}{\mathcal {L}_0} \end{aligned}$$

is the ratio of the likelihood $\mathcal {L}_0$ of the data given isotropically distributed UHECRs, and the likelihood $\mathcal {L}_\text {a}$ of the data given the alternative hypothesis simulated with PARSEC. In the $CL_S$ method, not *Q* directly, but the modified likelihood ratio

15 $$\begin{aligned} CL_S = \frac{P_\text {a}(Q \ge Q_\text {obs})}{1- P_0(Q\le Q_\text {obs})} \end{aligned}$$

is used as test statistic. Here $P_\text {a}(Q \ge Q_\text {obs})$ is the frequency with which likelihood ratios *Q* larger than the observed value are obtained in simulations of the alternative hypothesis and $1- P_0(Q\le Q_\text {obs})$ the corresponding frequency in simulations of the null hypothesis. Points in parameter space with $CL_S < 0.05$ are excluded at the 95 % confidence level. The resulting limits are shown in Fig. 6 for the individual observables.

A combination of the limits is not attempted here as it depends on scenario-specific correlations between the observables. If the cosmic rays are not protons but heavier nuclei the limits are reduced accordingly. For the extreme case that all cosmic rays are iron nuclei with $Z=26$ the limits shift down by more than one order of magnitude. For the proton case shown in Fig. 6 the extragalactic deflection of cosmic rays needs to be larger than $C_\text {E} =10-120^{\circ } \mathrm{Mpc}^{-1/2} \mathrm{Eev}$ for source densities smaller than $10^{-3} \,\mathrm{Mpc}^{-3}$ and assuming deflections in the galactic magnetic field as expected from the Jansson–Farrar 2012 model with a coherence length set to $L_\text {c} = 60\,\mathrm{pc}$. The exact value depends on the source density. Without galactic random field the limits are only marginally more constraining, choosing a higher coherence length lowers the limits according to the stronger deflections.

Previously, we derived from two-point correlations of UHECRs with an energy $E>60 \,\mathrm{EeV}$ lower bounds on the density of uniformly distributed sources of, e.g., $2 \times 10^{-4}\,\text {Mpc}^{-3}$ if the deflection of cosmic rays above 60 EeV is $5^{\circ }$ [12]. Only the total deflection due to the EGMF and GMF was taken into account, and no explicit model for the Galactic magnetic field was used. An approximate comparison with the current analysis can be performed assuming the average deflections in the EGMF and GMF add up linearly. The average deflection of 60 EeV cosmic rays in the JF2012 field accounts to $5^{\circ }$. The above density therefore gives a lower limit for negligible deflections in the EGMF.

With the current analysis we obtain for the lowest EGMF considered a limit of $9 \times 10^{-4}\,\text {Mpc}^{-3}$ from an analysis of the Thrust Minor. We therefore extend the lower bound on the density of uniformly distributed sources by a factor of more than four in the case of small extragalactic deflections.

## Conclusions

In this work, we characterized the distribution of UHECRs with $E >5 \,\mathrm{EeV}$ in regions of 0.25 rad around events with $E > 60 \,\mathrm{EeV}$ using observables sensitive to patterns characteristic for deflections in cosmic magnetic fields. No such patterns have been found within this analysis. We demonstrated the usage of this non-observation to constrain propagation scenarios using a scenario based on parametrizations for the propagation of UHECR protons as an example.

Within the simulated scenario, we estimate that the strength of the deflection in the extragalactic magnetic field has to be larger than $C_\text {E} = 10-120^{\circ } \mathrm{Mpc}^{-1/2} \mathrm{EeV}$ for source densities smaller than $10^{-3} \,\mathrm{Mpc}^{-3}$ assuming protons and deflections expected from the Jansson–Farrar 2012 model for the galactic magnetic field. For protons with an energy $E = 10 \,\mathrm{EeV}$ from a source at 16 Mpc this translates to a required strength of the deflection in extragalactic space of more than 4$^{\circ }$ if the source density is smaller than $10^{-3} \,\mathrm{Mpc}^{-3}$ and more than 25$^{\circ }$ if the source density is smaller than $10^{-4}\, \mathrm{Mpc}^{-3}$.
